# Data Sharing for Pediatric Clinical Trials Funded by the US National Institutes of Health

**DOI:** 10.1001/jamanetworkopen.2023.25342

**Published:** 2023-07-25

**Authors:** Claire Narang, Michelle Ouvina, Chris A. Rees, Florence T. Bourgeois

**Affiliations:** 1 Pediatric Therapeutics and Regulatory Science Initiative, Computational Health Informatics Program, Boston Children’s Hospital, Boston, Massachusetts; 2 Division of Pediatric Emergency Medicine, Emory University School of Medicine, Children’s Healthcare of Atlanta, Atlanta, Georgia; 3 Department of Pediatrics, Harvard Medical School, Boston, Massachusetts

## Abstract

This cross-sectional study assesses declared data sharing in publications for a recent set of pediatric clinical trials funded by the US National Institutes of Health (NIH).

## Introduction

The National Institutes of Health (NIH) has a longstanding commitment toward data sharing to maximize the value of clinical research. Sharing of individual participant data promotes transparency and trust in scientific findings, enables replication and validation of results, and facilitates data reuse in future studies. Most recently, effective January 25, 2023, the NIH began requiring data management and sharing plans in grant applications to increase sharing of scientific data generated from research supported by the NIH.^1^ Access to and reuse of data may be particularly valuable for pediatric research, because conducting clinical trials is often more challenging for pediatric populations.^2^ In this study, we assessed declared data sharing in publications for a recent set of pediatric clinical trials funded by the NIH.

## Methods

This cross-sectional study analyzed NIH-funded pediatric clinical trials with grants completed in 2017-2019 and with trial results published in a peer-reviewed publication by June 30, 2022. Methods for identification of trials enrolling pediatric patients (aged <18 years) and linkage to trial publications have been previously described.^3^ Each trial publication was reviewed by 2 authors to identify data sharing statements describing access to clinical trial data; any discrepancies were resolved by consensus. Information was collected on the type of data available and type of access provided. Journals were categorized according to affiliation with the International Committee of Medical Journal Editors (ICMJE), which has developed guidance and policies to promote data sharing for clinical trials and maintains a list of journals reporting compliance with ICMJE recommendations.^4,5^ Differences based on ICMJE affiliation were evaluated with 2-sided χ^2^ tests, with significance set at *P* < .05. The study followed STROBE reporting guidelines and was deemed exempt from review by the Boston Children’s Hospital institutional review board, as it relied on publicly available information.

## Results

Among 213 pediatric clinical trial publications, 69 (32.4%) included a data sharing statement and 62 (29.1%) declared that data were available (Table). For the 62 trials declaring data availability, the type of data shared consisted of individual participant data for 30 (48.4%) and was not specified for the remainder. Data were most frequently accessible through request to authors (43 [69.4%]), followed by request to a data repository (12 [19.4%]) or via a link to the data set (7 [11.3%]). Among the 12 trials declaring use of data repositories, data submission to the repository could be verified for 8 (66.7%). Overall, among the 213 trial publications, 7 (3.3%) provided individual participant data that could be readily accessed.

**Table.** Data Sharing for National Institutes of Health–Funded Pediatric Clinical Trials

Characteristic	No./total No. (%)			*P* value
	Total (N = 213)	Published in ICMJE-affiliated journal (n = 75)	Published in non–ICMJE-affiliated journal (n = 138)	
Data declared as available, No. (%)				
Yes	62 (29.1)	26 (34.7)	36 (26.1)	.19
No	151 (70.9)	49 (65.3)	102 (73.9)	
Type of data available				
Individual participant data	30/62 (48.4)	14/26 (53.8)	16/36 (44.4)	.46
Unspecified	32/62 (51.6)	12/26 (46.2)	20/36 (55.6)	
Type of data access				
Available upon request to author	43/62 (69.4)	19/26 (73.1)	24/36 (66.7)	.59
Available upon request to repository	12/62 (19.4)	6/26 (23.1)	6/36 (16.7)	.53
Retrievable for downloading and use	7/62 (11.3)	1/26 (3.8)	6/36 (16.7)	.12

Abbreviation: ICMJE, International Committee of Medical Journal Editors.

Publications in ICMJE-affiliated journals were not more likely to provide a data sharing statement than those in non–ICMJE-affiliated journals (29 of 75 [38.7%] vs 40 of 138 [29.0%]; *P* = .15). Similarly, data availability and type of data access did not differ according to ICMJE affiliation.

## Discussion

This study’s findings suggest that data for most NIH-funded pediatric clinical trials were not readily available for reuse. These findings are consistent with prior studies indicating that access to individual trial participant data remains limited, even among trials published in ICMJE-affiliated journals.^5^ Limitations of the study include potential lack of generalizability to trials studying adults and those funded by non-NIH entities. We also were not able to confirm data sharing for trials requiring data requests, which could have resulted in overestimation of actual data availability.^6^

This analysis provides a benchmark for future assessments of clinical trial data sharing as the NIH’s new policy goes into effect. Such evaluations should include specific measures to capture data sharing for patient populations that may have more limited clinical trial data, including pediatric patients or those with rare diseases. Furthermore, monitoring activities should focus not only on data availability and ease of access but also on measuring effective data reuse and benefits of secondary studies in advancing scientific knowledge.

## Notes

Supplement.
Data Sharing Statement
