# Supplementary material for: Data Sharing for Pediatric Clinical Trials Funded by the US National Institutes of Health
Source: JAMA Netw Open. 2023 Jul 25;6(7):e2325342. doi: 10.1001/jamanetworkopen.2023.25342 (PMC10370255; doi:10.1001/jamanetworkopen.2023.25342)
Supplement: Supplement. — Data Sharing Statement [file jamanetwopen-e2325342-s001.pdf]

Data Sharing Statement

Narang. Data Sharing for Pediatric Clinical Trials Funded by the US National Institutes of Health. *JAMA Netw Open*. Published July 25, 2023. doi:10.1001/jamanetworkopen.2023.25342

Data
Data available: Yes
Data types: Data (not involving human participants)
How to access data: All study data are available in Figshare at the following link: https://doi.org/10.6084/m9.figshare.23608797.v1
When available: With publication

Supporting Documents
Document types: None

Additional Information
Who can access the data: Anyone requesting the data
Types of analyses: For any purpose
Mechanisms of data availability: The data will be made available to all researchers without prior approval.
